# Viewpoint: how to measure comorbidities in patients with rheumatoid arthritis – clinical and academic value

**DOI:** 10.1093/rheumatology/kead436

**Published:** 2023-10-23

**Authors:** Helga Radner

**Affiliations:** Division of Rheumatology, Department of Internal Medicine III, Medical University Vienna, Vienna, Austria

**Keywords:** comorbidity, multimorbidity, rheumatoid arthritis, burden of disease

## Abstract

Given the high prevalence and the enormous impact on key outcomes, comorbidities are important to consider, especially in patients with RA. Comorbidity indices are tools to quantify the impact of the overall burden of coexisting diseases on a specific outcome of interest. Until now, no gold standard exists on how to measure comorbidities. A large variety of indices have been developed using different settings and therefore leading to conceptual differences. Choosing the right tool clearly depends on the intention (clinical or research purpose) and the specific research question. The current article will address the purpose and challenge of measuring comorbidities in RA patients.

Rheumatology key messages

- Comorbidities have an enormous impact on important outcomes in RA patients.
- Standardized measurement of comorbidities is important; however, no gold standard exists.

## Introduction

Comorbidities are highly prevalent in rheumatic conditions. Over 60% of patients with rheumatoid arthritis (RA) are considered to be multimorbid, with an average of two comorbid conditions per patient in addition to RA [1, 2]. Consideration of comorbidities is important from a clinical as well as a research perspective.

Comorbidities have a large impact on important disease-specific and overall outcomes [3–6]. Comorbidities are associated with higher mortality rates and premature death [7]. RA patients with comorbidities have poorer quality of life and are more severely physically impaired [4, 5]. Independent of RA disease activity, functional ability deteriorates with the increasing number of comorbid conditions [3]. Comorbid RA patients are less likely to achieve remission [8], which could be explained by different factors. Studies have shown less intensive treatment of RA than indicated in patients with comorbidities, probably due to fear of polypharmacy, adverse events or a general tendency to undertreat older, and therefore more likely multimorbid, individuals [9–12]. Further, there seems to be mitigated treatment response in multimorbid patients [5, 13]. Clinical care of multimorbid patients is challenging. As patients with significant comorbidities are typically excluded from randomized controlled trials, only limited evidence on the efficacy and safety of DMARDs exists. Further, management recommendations and guidelines are developed for single disease only, based on evidence gained in such randomized controlled trials (RCTs), neglecting the presence of multimorbidity.

Although a significant number of studies on comorbidity and its impact on RA patients exist, systematic comparison of studies or meta-analyses of the effect of comorbidities on certain outcomes are lacking. Such efforts are hampered by lack of standardization in how to assess and quantify comorbidities. The reasons for this are varied. First of all, conceptual differences of being afflicted by more than one condition exit [1], leading to development of comorbidity in contrast multimorbidity indices. Second, the heterogeneity of comorbidities with a large variety of severity, complexity and impact on a patient complicates creation of the comorbidity index. Further, existing comorbidity indices were developed and applied in different settings, addressing different research or clinical questions.

In the following article, conceptual differences and differences in development and application of various indices are discussed.

### Measuring comorbidity and multimorbidity

Even though the concepts of comorbidity and multimorbidity are not contradictory or mutually exclusive, they use different perspectives of being afflicted with more than one condition. While the concept of comorbidity is based on a single index disease with co-existing diseases of less importance, in the concept of multimorbidity co-occurring diseases are regarded as being of equal importance. These conceptual differences also lead to differences in indices assessing the underlying concept. While multimorbidity indices are often more generic and therefore used in primary or acute care settings, comorbidity indices are based on a clearly defined index disease, developed and validated in cohorts with certain morbid conditions. For example, the Charlson Comorbidity index (CCI), even though regarded as a generic comorbidity assessment tool, was initially developed in a breast cancer cohort [14]. Only a limited number of comorbidity indices were developed or at least validated in RA cohorts [15, 16].

Table 1 gives an overview of the most prominent generic and frequently used indices in rheumatology as identified in literature [2, 17, 18]. Existing tools differ in number and type of condition included. One may include any existing comorbidity while others may focus on specific and pre-selected conditions. Based on a review, number of morbidities included varies from 4 to 102 different conditions [19]. Depending on the index, selection of morbidities might be based on expert opinion, prevalence of comorbidities or impact on certain outcomes of interest. Authors of a systematic review on different comorbidity measurement tools have concluded that most studies (60%) have stated no clear selection criteria [19]. Inclusion is often based on pragmatic reasons such as availability of data or expected prevalence of certain conditions. In contrast, item generation for the Functional Comorbidity Index (FCI) was based on literature investigating impact on physical function combined with input gained from focus groups of clinicians and patients [20]. Similarly, the Multimorbidity Index (MMI) includes 40 main chronic comorbid conditions identified by systematic literature covering all body systems, reviewed and discussed by experts [15].

**Table 1.** Overview of common comorbidity indices

Index	Population	Data source	Morbidities (*n*)	Weighting	Predicted outcome to test validity
Charlson Comorbidity Index [14]	Hospital breast cancer cohort	Medical records	11	Y	Mortality
Chronic disease score [34]	General population	Administrative data	22	Y	Mortality; hospitalization
Elixhauser Comorbidity Index [21]	Acute care patients	Administrative data	30	N	Length of stay/Hospital charge/in-hospital mortality
Functional Comorbidity Index [20]	Patients seeking spine aliment	Self-reported	27	N	Physical function
Index of co-existing disease [35]	Patient undergoing hip replacement	Medical records	14	Y	Post-operative complication; functional limitation
Medication Based Burden Index [36]	Hospital patient	Medication/Administrative data	20	Y	Mortality/re-admission
Multimorbidity Index [15]	Rheumatoid arthritis patients	Medical records	40/12	Y/N	Health related quality of life
Kaplan–Feinstein Index [37]	Male veterans with diabetes	Medical records	13	Y	Mortality
Rheumatic Disease Comorbidity Index [16]	Rheumatic disease cohort	Physician/self-reported	11	Y	Mortality; physical function; costs

After identification of adequate number and type of comorbidities, one needs to decide on whether to treat them all equally or assign different weightings. The simplest way of assessment is a count of co-existing conditions per patients. This implies equal importance and impact of co-existing diseases, independent on severity or relevance. When predicting mortality, the impact of chronic kidney disease will differ according to stage of disease. Similarly, the impact of myocardial infarction might be higher as compared with glaucoma. Therefore, a large number of common comorbidity indices have assigned specific weights, depending on the perceived or calculated impact of each comorbid condition on distinct outcomes. Frequently used outcomes include mortality, length of stay in hospital, physical function or health-related quality of life (HRQoL). Weightings can be based on complex models, calculated from hazard ratios or regression coefficients. For example, comorbidities included in the CCI are weighted according to relative mortality risk, assigning the highest score to severe conditions such as AIDS or metastatic cancer [14]. Other approaches to assign weights are based on self-reported severity or perceived burden of disease. Depending on methodology and outcome of interest, weighting of specific conditions can vary across different scores.

Besides deciding on number and type of comorbidities included, indices also vary in terms of data source used to collect comorbidities. Measurement tools can be based on information gained via patients’ self-reports [16, 20], medical records and physician reports [15] or administrative data [15, 21]. The obtained information can further be extracted using codes such as the international classification of disease [22] or via prescribed medication. Each data source has both advantages and disadvantages: using the right population, self-reporting of comorbidities can be simple and time saving, and sometimes the only option in case no charts are available. However, there is the risk of recall or reporting bias. It has been shown that certain diseases such as RA might be overestimated as patients cannot differentiate between RA, osteoarthritis or other arthritic conditions [23]. Factors associated with the decreasing quality of self-reported data are increasing age, number of morbid conditions or lower level of education [24]. Identifying comorbidities by chart review outperforms data collection using other sources such as administrative data [25]. However, it might be cumbersome, time and resource consuming. Use of administrative data is time efficient and especially beneficial for large data sets; however, it relies on availability and quality of data due to incorrect or missing data.

### Reliability and validity of different tools to measure comorbidities

*Reliability* refers to the consistency of a certain measurement. This is of special importance when scores are calculated by different people, at different times or by using different data sources. Several indices have proven a high level of agreement using self-administered questionnaires compared with administrative data sources [26]. Further, scores like the CCI or the self-administered comorbidity questionnaire have shown moderate to high test-retest intra-class correlation [27, 28].

*Validity* refers to what extent a measurement represents what it intends to measure. A valid comorbidity index should demonstrate high performance covering several aspects of validity, as given below.

*Content validity* refers to the completeness and relevance of the content of a measurement tool. This is linked to selection and weighting of comorbidities included in the specific tool. Selection and weighting of comorbidities included in the CCI is based on regression models predicting mortality. However, if CCI is used to calculate the impact of comorbidities on other important outcomes such as HRQoL, physical function or disease activity in RA, relevant comorbidities are missing. Highly prevalent comorbidities of RA patients such as depression, obesity or fibromyalgia shown to be associated with poor outcomes are lacking [29, 30]. Other indices such as MMI, FCI or Rheumatic Disease Comorbidity Index (RDCI) have been developed and validated specifically in patients with rheumatic conditions such as RA. The predictive ability of measurement tools is strongly associated with the property of content validity. Therefore, it is obvious that indices such as FCI or MMI, developed to predict specific outcomes like physical function or HRQoL will outperform other indices and show higher content validity within a certain context. Only a few studies have directly compared performance of different indices in RA patients. In one study MMI shows higher performance in prediction of HRQoL, fatigue or physical function compared with CCI and FCI [15]. In contrast, CCI and RDCI seem to be slightly better in predicting mortality compared with MMI or FCI [16–18].

*Criterion validity* compares the performance of an index to another valid measurement, ideally the accepted gold-standard in the field. As there is no agreement on a gold-standard of how to measure comorbidities, criterion validity is often demonstrated by comparison with other existing indices. Due to the above explained differences of comorbidity indices, data on criterion validity are inconsistent.

*Construct validity* refers to the extent of how a scale performs under various conditions, for example using different diseases or patient populations (i.e. hospitalized patients or elderly patients). Usually, comorbidity indices were developed in a specific population, with only a minority in patients with rheumatic conditions and even less in RA patients. However, several studies have shown good performance of comorbidity indices when used in a different patient population than in which they were initially developed [31].

## Choosing the right tool to measure comorbidities

Considering the large impact of comorbidities on various outcomes, there is a clear need to measure comorbidities. In order to select the right tool, it is necessary to consider the intention: *why* you want to assess comorbidities*.* From a clinical perspective it is important to quantify the burden of illness and to take into account the complexity of multimorbidity in individual management strategies. This requires a comprehensive comorbidity assessment on a regular basis. However, feasibility and time constraints or lack of information on what and how to screen comorbidities hampers such efforts. Several years ago, EULAR provided a checklist for screening and reporting comorbidities in inflammatory arthritis [32], but the exhaustive list may again pose a challenge for implementing this in a tightly scheduled clinical routine. Existing comorbidity indices were developed for research purposes, transforming a holistic concept of burden of illness into a single numeric score. For routine care, such indices are typically too coarse and insensitive to replace clinical judgment, but also for research purposes, feasibility clearly differs across various indices. While MMI includes a rather comprehensive list of 40 different morbidities, RDCI or CCI include only 11 different conditions. Further availability of data sources clearly limits feasibility. Self-report of comorbidity is only feasible in prospective studies, and recommended to severe and surgical conditions only [33]. To calculate indices using administrative data such as the Elixhauser Comorbidity Index, coding can help to identify morbidities within large data sets and therefore increase feasibility. However, one needs to take into account country-specific codings, coding errors or reporting bias. In contrast, indices based on chart review might show better performance; however, these are time-consuming and therefore lack feasibility [25].

As no gold standard exists, selecting the right measurement tool for research purposes clearly depends on the study question. Existing indices were developed for specific aims in different settings and therefore index performance clearly depends on the outcome of interest, study population and the index used. Further, it depends on available data source (i.e. administrative data *vs* self-administered questionnaire) and on quality and completeness of data. Investigators have to take these aspects into account when deciding on the right index. Indices such as the RDCI and MMI have been developed in a population of patients with rheumatic conditions and tested in RA. It is unclear yet whether inclusion of specific morbidities is essential for specific research questions or whether more generic tools are valid enough. Further studies are needed to compare indices in different research settings. This will be necessary to identify and agree on the optimal index, which would further allow comparability of impact of comorbidities across studies.

## Data Availability

There are no new data associated with this article.
